# Multimodality Imaging‐Guided Pulsed Field Ablation for Atrial Fibrillation in Cor Triatriatum Sinister

**DOI:** 10.1002/joa3.70474

**Published:** 2026-09-25

**Authors:** Kazuya Murata, Yasuteru Yamauchi, Atsuhito Oda, Hirofumi Arai, Tetsuo Sasano

**Affiliations:** ^1^ Department of Cardiology Japan Red Cross Yokohama City Bay Hospital Yokohama City Kanagawa Japan; ^2^ Department of Cardiovascular Medicine Institute of Science Tokyo Bunkyo‐ku Tokyo Japan

## Abstract

Multimodality imaging facilitated safe transseptal access and catheter navigation in a patient with cor triatriatum sinister. CARTO‐integrated VARIPULSE pulsed‐field ablation achieved pulmonary vein isolation without complications, demonstrating a feasible treatment strategy for atrial fibrillation in complex congenital left atrial anatomy.

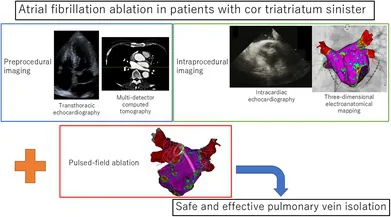

Cor triatriatum sinister (CTS) is a rare congenital cardiac anomaly, in which a fibromuscular membrane divides the left atrium into two chambers; it accounts for approximately 0.1% of congenital heart disease [1]. CTS can be associated with hemodynamic disturbance, heart failure, and atrial fibrillation (AF). The clinical presentation of CTS depends on the degree of membrane obstruction. In a systematic review of adults with CTS, obstructive physiology was found to be associated with younger age at diagnosis and more frequent congestive heart failure and pulmonary hypertension, whereas AF was the most common manifestation, occurring in 32.8% of patients [2]. Patients with nonobstructive CTS may remain asymptomatic until adulthood and be diagnosed incidentally or during evaluation for atrial arrhythmias; however, catheter ablation for AF has rarely been reported [3]. Given the complex anatomy and frequent coexistence of other congenital anomalies, careful multimodality imaging is essential for safe ablation. Herein, we report a case of AF in a patient with CTS in whom pulmonary vein (PV) isolation was safely achieved using multimodality imaging‐guided pulsed‐field ablation.

A 76‐year‐old man with a history of catheter ablation for atrial flutter (16 years earlier) and percutaneous coronary intervention in ischaemic heart disease (2 years earlier) was referred for catheter ablation due to symptomatic paroxysmal AF. Transthoracic echocardiography revealed an enlarged left atrium (LA), measuring 46 mm in diameter, divided into two chambers by a membrane, consistent with Lucas‐Schmid type Ia CTS (Figure 1). No pressure gradient was detected between the dorsal accessory chamber (AC) and ventral main chamber (MC). Left ventricular ejection fraction was preserved at 68%.

**FIGURE 1:**
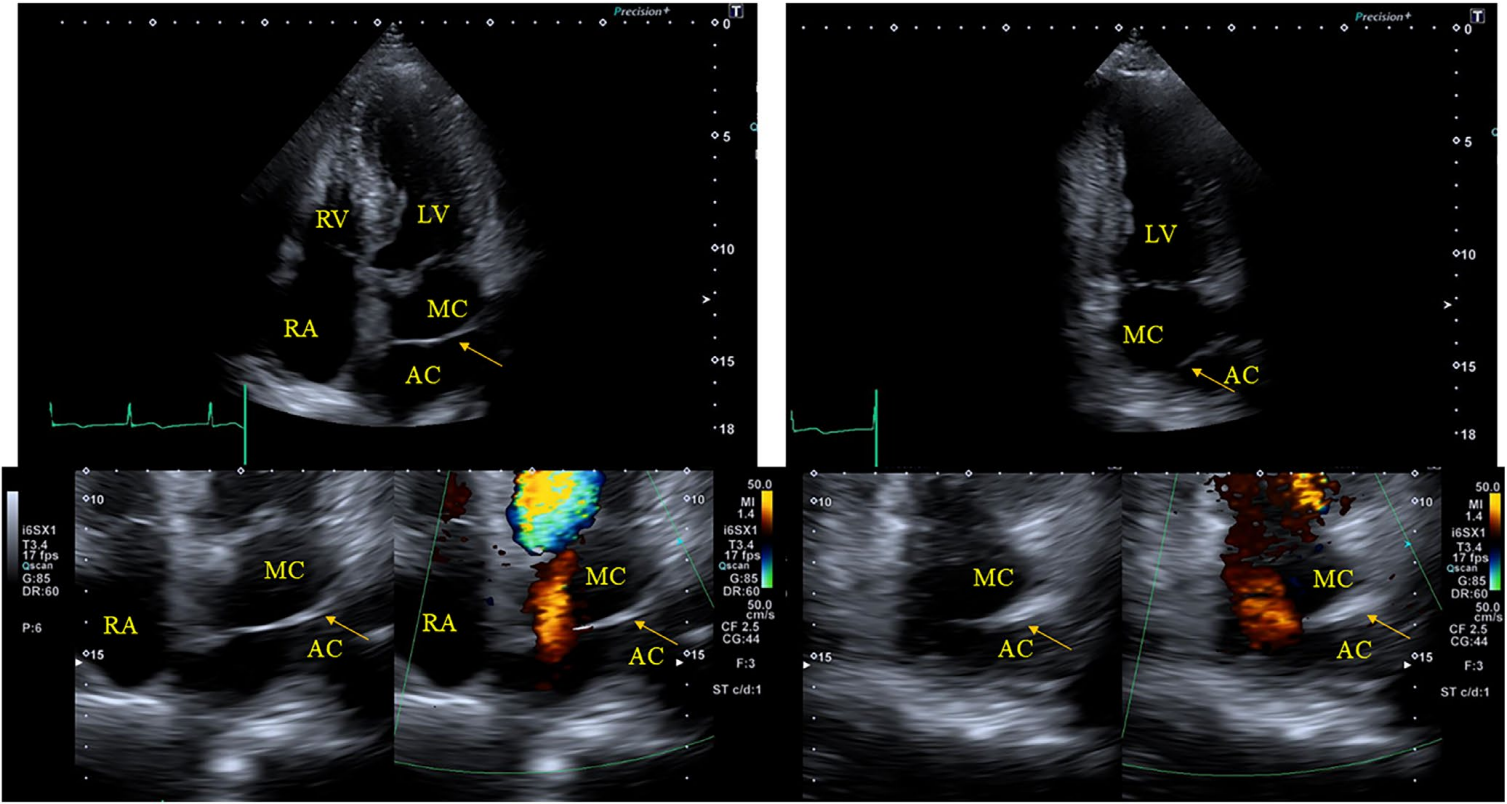
Echocardiographic findings. Transthoracic echocardiography demonstrates the division of the left atrium (LA) into an accessory chamber (AC) and a main chamber (MC) by a fibromuscular membrane. A discrete fenestration is visible within the membrane (arrowheads). AC, accessory chamber; LA, left atrium; LV, left ventricle; MC, main chamber; RA, right atrium; RV, right ventricle.

Multi‐detector computed tomography (MDCT) confirmed that the membrane extended from near the fossa ovalis (FO) toward the Coumadin ridge and posterior‐inferior left atrial wall (Figure 2a). Two openings were detected in the membrane at the inferior septal aspect of the atrium, one of which had a tunnel‐like morphology (Figure 2b,c). All PVs drained into the AC, whereas no left atrial thrombus or other congenital cardiac anomalies were detected. As the FO provided a direct route to the AC, transseptal access was planned using intracardiac echocardiography (ICE), three‐dimensional electroanatomical mapping, and CT image integration.

**FIGURE 2:**
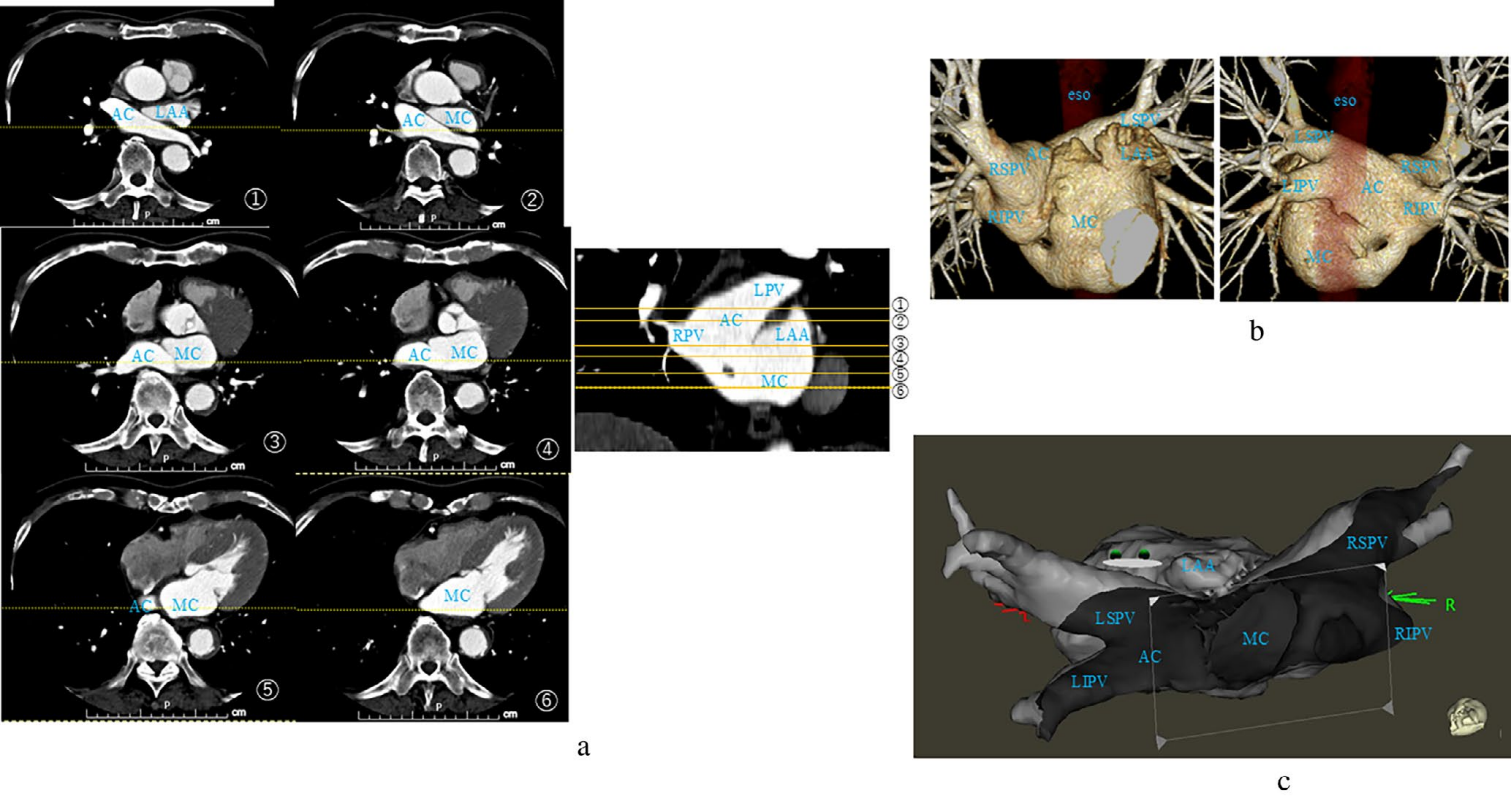
Multi‐detector computed tomography (MDCT) findings. (a) Coronal MDCT image of the LA (right) with corresponding numbered axial sections (left). (b) Three‐dimensional reconstruction showing all pulmonary veins (PVs) draining into the AC. (c) Three‐dimensional reconstruction with a superior inner view demonstrates that the LA is partitioned by a membrane containing two fenestrations. AC, accessory chamber; LA, left atrium; LAA, left atrial appendage; LS, left superior; LI, left inferior; MC, main chamber; PV, pulmonary vein; RS, right superior; RI, right inferior.

Under deep sedation, the LA and FO were reconstructed using CARTOSOUND (Biosense Webster, Diamond Bar, CA, USA) and integrated with CT images. Transseptal puncture was performed through an 8.5‐Fr VIZIGO sheath (Biosense Webster) under ICE guidance, merged LA MDCT images, and CARTO UNIVIEW (Biosense Webster) guidance, enabling accurate cannulation of the AC while avoiding membrane injury (Figure 3a–c). On the LA side, the membrane was further visualized using ICE (Figure 3d). Contrast injection and high‐density mapping with OCTARAY (Biosense Webster) confirmed the anatomical relationship between the MC and AC and the location of all PVs (Figure 3e).

**FIGURE 3:**
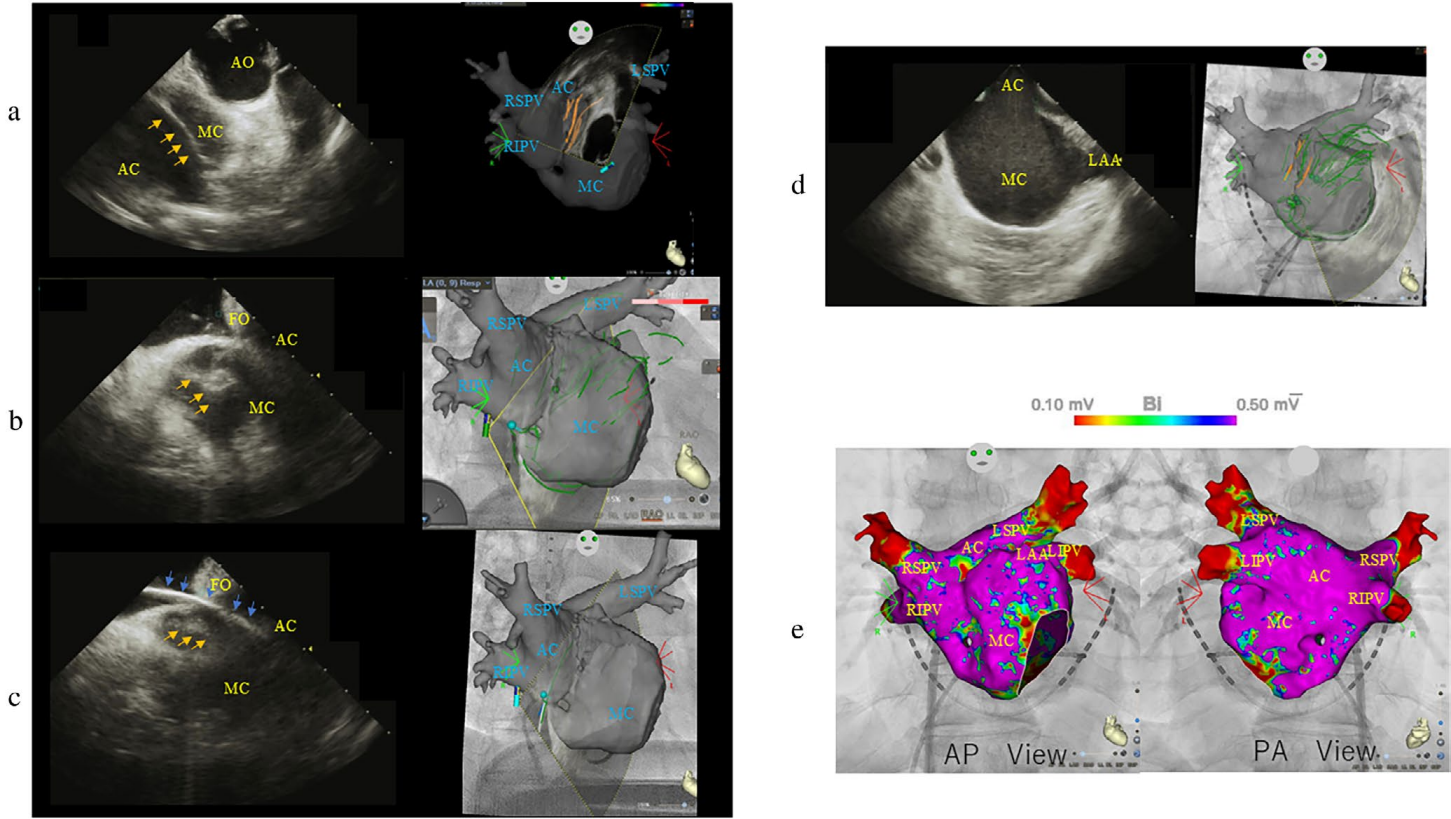
Intracardiac echocardiography (ICE) and image integration. (a) ICE view of the right ventricular outflow tract toward the LA. The LA and membrane (orange arrowheads) were mapped from the right ventricular outflow tract and RA and then merged with the CT‐derived anatomy. (b) Before transseptal puncture, the transseptal needle tens the fossa ovalis toward the AC. (c) After transseptal puncture, the guidewires (blue arrowheads) were advanced into the AC through the fossa ovalis (FO). (d) After sheath entry into the LA, additional ICE imaging was performed from the LA side to refine the merged image. (e) LA voltage maps during RA pacing. Anteroposterior (AP) and posteroanterior (PA) views are overlaid on the LA angiogram. The voltage was color‐coded from red (low) to purple (normal). AC, accessory chamber; AO, aorta; AP, anteroposterior; FO, fossa ovalis; ICE, intracardiac echocardiography; LA, left atrium; LAA, left atrial appendage; LS, left superior; LI, left inferior; MC, main chamber; PA, posteroanterior; PV, pulmonary vein; RA, right atrium; RS, right superior; RI, right inferior.

A VARIPULSE pulsed‐field ablation catheter (Biosense Webster) was advanced into the AC through the VIZIGO sheath. PV isolation was performed at the antra of both ipsilateral PVs, with confirmed elimination of PV potentials and bidirectional conduction block (Figure 4). Cavotricuspid isthmus ablation was subsequently performed using an irrigated radiofrequency catheter. Isoproterenol provocation revealed no non‐PV triggers. The procedure was completed without complications. At the 1‐year follow‐up, the patient remained free of AF.

**FIGURE 4:**
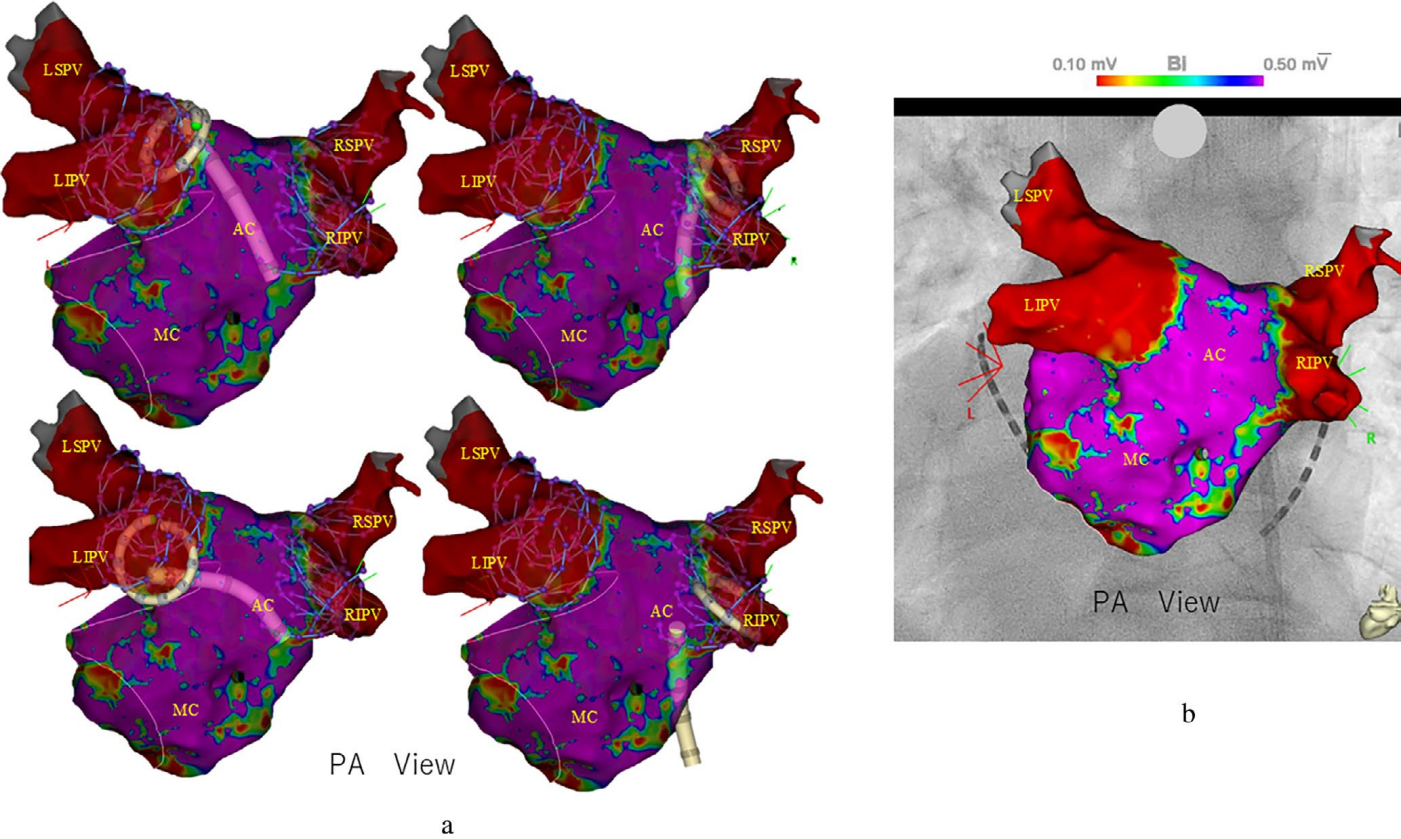
Post‐ablation voltage maps. (a) Post‐pulsed‐field ablation (PFA) LA voltage map. Purple dots and lines indicate the PFA application sites. The positions of the VARIPULSE catheter and sheath during ablation of each PV are annotated. The voltage was color‐coded from red (low) to purple (normal). (b) The PA‐view LA voltage map during RA pacing after PFA was overlaid on the LA angiogram. AC, accessory chamber; LA, left atrium; LS, left superior; LI, left inferior; MC, main chamber; PA, posteroanterior; PFA, pulsed‐field ablation; PV, pulmonary vein; RA, right atrium; RS, right superior; RI, right inferior.

In the absence of significant obstruction, AF in this patient may have primarily reflected age‐related atrial remodeling, consistent with previous reports. However, congenital left atrial abnormalities still require careful anatomical assessment during AF ablation [3]. CTS poses unique challenges for catheter ablation [3] because a fibromuscular membrane divides the LA and can complicate transseptal access, catheter navigation, and PV isolation. In the present case, multimodality imaging with MDCT, ICE, and three‐dimensional electroanatomical mapping was essential for understanding the anatomical relationships among the FO, AC, MC, membrane, and PVs. This strategy enabled safe transseptal puncture and controlled catheter manipulation within the AC.

In this case, the main chamber was mildly enlarged, with only limited patchy low‐voltage areas and no extensive left atrial substrate. Direct mapping of the thin, mobile, and fenestrated membrane was limited; however, no abnormal or fractionated electrograms suggesting an arrhythmogenic substrate were identified. As previous reports have linked the membrane attachment to low‐voltage areas and macro‐reentrant atrial tachycardia, we specifically assessed this region for non‐PV triggers [3]. During isoproterenol provocation (10 μg), AF was induced once by atrial burst pacing; however, after electrical cardioversion, no reproducible non‐PV triggers or sustained atrial tachyarrhythmias were observed. Therefore, no ablation beyond pulmonary vein isolation and cavotricuspid isthmus ablation was performed.

The VARIPULSE system was selected because of several features that were considered advantageous in this anatomically complex case. First, its compatibility with an 8.5‐Fr steerable sheath allowed pulmonary vein isolation through a single, relatively small transseptal access, which may reduce procedural complexity and the potential risk of membrane injury in CTS. Second, because the pulmonary vein antra appeared relatively large, the variable‐loop design of the VARIPULSE catheter was considered suitable for achieving wider antral pulmonary vein isolation while adapting to the anatomy of the accessory chamber. Third, the VARIPULSE system is integrated with the CARTO system, enabling seamless CT image integration, three‐dimensional electroanatomical mapping, and precise catheter visualization. Moreover, the impedance‐based tissue proximity indicator provided real‐time information regarding catheter–tissue proximity, which was useful for assessing catheter positioning and tissue contact within the accessory chamber and may have contributed to efficient ablation with reduced fluoroscopic guidance [4]. These features may be particularly useful in patients with congenital left atrial anomalies, in whom anatomical orientation and controlled catheter manipulation are essential.

Although successful zero‐fluoroscopy PFA for AF in CTS has been previously reported [5], the use of the VARIPULSE system in this setting has not been described. Thus, the present case demonstrates the feasibility and safe strategy of VARIPULSE‐guided PFA supported by multimodality imaging and tissue proximity assessment in a patient with CTS.

## Funding

The authors have nothing to report.

## Consent

The authors confirm that written consent for the submission and publication of this case report, including images and associated text, has been obtained from the patient in line with COPE guidance.

## Conflicts of Interest

The authors declare no conflicts of interest.

## Data Availability

The data that support the findings of this study are available from the corresponding author upon reasonable request.

## References

[1] S. Yaroglu Kazanci , S. Emani , and D. B. McElhinney , “Outcome After Repair of Cor Triatriatum,” American Journal of Cardiology 109 (2012): 412–416. 10.1016/j.amjcard.2011.09.029 22078218

[2] V. Rudienė , C. M. S. Hjortshøj , S. Glaveckaitė , et al., “Cor Triatriatum Sinister Diagnosed in the Adulthood: A Systematic Review,” Heart 105 (2019): 1197–1202. 10.1136/heartjnl-2019-314714 31171629

[3] A. Karimianpour , A. W. Cai , F. A. Cuoco , J. L. Sturdivant , S. E. Litwin , and J. M. Wharton , “Catheter Ablation of Atrial Fibrillation in Patients With Cor Triatriatum Sinister: Case Series and Review,” Pacing and Clinical Electrophysiology 44 (2021): 2084–2091. 10.1111/pace.14371 34648196

[4] S. Hirata , K. Nagashima , R. Watanabe , et al., “Workflow of the Zero‐Fluoro Pulsed Field Ablation,” Journal of Arrhythmia 40 (2024): 1529–1532. 10.1002/joa3.13174 PMC11632259 39669927

[5] Y. Mizuno , Y. Munehisa , D. Kumazawa , K. Onodera , K. Satomi , and K. Yamashita , “Zero‐Fluoroscopy Pulsed Field Ablation of Atrial Fibrillation in a Patient With Cor Triatriatum Sinister: A Case Report,” HeartRhythm Case Reports 12 (2026): 426–431. 10.1016/j.hrcr.2026.01.002 PMC13100610 42028073

